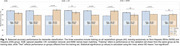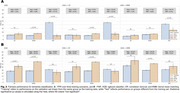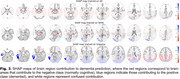# Addressing AI Bias in MRI‐Based Dementia Classification Among Hispanic Individuals

**DOI:** 10.1002/alz70856_105856

**Published:** 2026-01-08

**Authors:** Ngoc‐Huynh Ho, Sokratis Charisis, Sachintha Ransara Brandigampala, Di Wang, Susan R. Heckbert, David M Martinez, Timothy M. Hughes, Nicolas Honnorat, Derek B. Archer, Timothy J. Hohman, Sudha Seshadri, Christos Davatzikos, Mohamad Habes

**Affiliations:** ^1^ Glenn Biggs Institute for Alzheimer's & Neurodegenerative Diseases, University of Texas Health Sciences Center at San Antonio, San Antonio, TX, USA; ^2^ University of Texas Health Science Center at San Antonio, San Antonio, TX, USA; ^3^ UT Health Science Center at San Antonio, San Antonio, TX, USA; ^4^ University of Washington, Seattle, WA, USA; ^5^ Wake Forest University School of Medicine, Winston‐Salem, NC, USA; ^6^ Vanderbilt Genetics Institute, Vanderbilt University Medical Center, Nashville, TN, USA; ^7^ Vanderbilt Memory & Alzheimer's Center, Vanderbilt University Medical Center, Nashville, TN, USA; ^8^ Department of Neurology, Vanderbilt Memory & Alzheimer's Center, Vanderbilt University Medical Center, Nashville, TN, USA; ^9^ Department of Neurology, Vanderbilt University Medical Center, Nashville, TN, USA; ^10^ Vanderbilt Brain Institute, Vanderbilt University Medical Center, Nashville, TN, USA; ^11^ Vanderbilt Memory and Alzheimer's Center, Vanderbilt University School of Medicine, Nashville, TN, USA; ^12^ Glenn Biggs Institute for Alzheimer's and Neurodegenerative Diseases, University of Texas Health Science Center, San Antonio, TX, USA; ^13^ Department of Radiology, University of Pennsylvania, Philadelphia, PA, USA; ^14^ Glenn Biggs Institute for Alzheimer's & Neurodegenerative Diseases, University of Texas Health Sciences Center at San Antonio, San Antonio, TX, USA

## Abstract

**Background:**

MRI based dementia classification using diagnostic tools trained predominantly on non‐Hispanic White (NHW) participants often fail to generalize to other groups such as Hispanic, leading to biased outcomes. This study investigates disparities in dementia classification between NHW and Hispanic populations and evaluates bias mitigation techniques to improve diagnostic fairness.

**Method:**

MRI‐based features without harmonization were extracted using MUSE for 2,541 NHW and 249 Hispanic participants from the National Alzheimer's Coordinating Center for classifying normally cognitive and demented patients, with dementia prevalences of 35.97% and 45.78%, respectively. XGBoost classifiers were trained and assessed using balanced accuracy (BA) and fairness metrics, including false positive rate (FPR) and false negative rate (FNR) disparities. Bias mitigation techniques such as correlation removal (CR) and Kernel Mean Matching (KMM) were implemented. SHAP maps illustrated population‐specific brain regions contributing to dementia predictions.

**Result:**

KMM demonstrated the lowest disparity in BA between NHW and Hispanic populations (Figure 1), with differences of 1.5% and 6% when trained on Hispanic and NHW datasets, respectively. CR slightly enhanced overall model accuracy when trained on the combined dataset, achieving a 0.8% improvement over the baseline, and reduced disparities to 4%. KMM minimized FPR differences, achieving 0.8% when trained on NHW and 8.3% when trained on Hispanic data, and recorded the lowest FNR disparity of 11.2% for NHW training (Figure 2). CR achieved the smallest FPR differences when trained on all groups and the lowest FNR disparity when trained on Hispanic participants. SHAP visualizations (Figure 3) revealed that the model identified population‐specific structural brain changes related to dementia, such as hippocampal and temporal lobe importance in NHW and occipital lobe contributions in Hispanic participants. These findings emphasize the risk of biased predictions when models are trained on a single population and tested on another.

**Conclusion:**

This study highlights the need for inclusive training datasets and robust bias mitigation strategies to address disparities in MRI based dementia classification across NHW and Hispanic populations. Techniques such as KMM and CR effectively reduced performance gaps, though balancing fairness with accuracy remains a challenge. The findings encourage robust strategies to enhance fairness in AI‐based healthcare diagnostics.